# The Glymphatic System and Diaphragmatic Dysfunction in Patients with Chronic Obstructive Pulmonary Disease and Chronic Heart Failure: The Importance of Inspiratory Rehabilitation Training

**DOI:** 10.3390/jcdd12100390

**Published:** 2025-10-02

**Authors:** Bruno Bordoni, Bruno Morabito, Vincenzo Myftari, Andrea D’Amato, Paolo Severino

**Affiliations:** 1 Department of Cardiology, Foundation Don Carlo Gnocchi IRCCS, Institute of Hospitalization and Care with Scientific Address, S Maria Nascente, Via Capecelatro 66, 20100 Milan, Italy; bordonibruno@hotmail.com; 2 Faculty of Medicine, U.C.S.C. Agostino Gemelli University Hospital Foundation, Largo Agostino Gemelli 8, 00136 Rome, Italy; brunomorabito84@gmail.com; 3 Department of Clinical, Internal, Anesthesiology and Cardiovascular Sciences, Sapienza University of Rome, Viale del Policlinico 155, 00161 Rome, Italy; vincenzo.myftari@gmail.com (V.M.); paolo.severino@uniroma1.it (P.S.)

**Keywords:** chronic obstructive pulmonary disease, chronic heart failure, inspiratory muscle training, diaphragm, rehabilitation, glymphatic system, cognitive decline

## Abstract

Chronic obstructive pulmonary disease (COPD) and chronic heart failure (CHF) are pathologies that impact mortality and morbidity worldwide. These chronic diseases have multiple causes, and they share some common clinical symptoms, such as diaphragm dysfunction (DD) and cognitive decline (CD), which, in turn, increase the mortality and morbidity rates in patients with COPD and CHF. One of the causes of CD is impaired glymphatic system function, with an accumulation of proteins and metabolites in the central nervous system. The glymphatic system is a structure that has not yet been widely considered by researchers and clinicians. Three key factors stimulate the ongoing physiological function of the glymphatic system: autonomic balance, heart rate, and, most importantly, the diaphragm. All these factors are altered in patients with COPD and CHF. This article reviews the relationship between the importance of the diaphragm, the glymphatic system, and CD, focusing on inspiratory rehabilitation training (IMT). Based on the data reported in this narrative review, we can strongly speculate that a consistent regimen of IMT in patients can improve cognitive status, reducing the cascade of symptoms that follow the diagnosis of CD. Further research is needed to understand whether targeting the glymphatic system with IMT is an effective option for helping patients delay the onset of CD.

## 1. Introduction

There are several chronic diseases that contribute to increasing the mortality and morbidity rates. Chronic obstructive pulmonary disease (COPD) is caused by non-physiological alterations and remodeling of the lung structures (from the parenchyma to the alveolus), which lead to progressive airway obstruction [1]. The Global Strategy for Prevention, Diagnosis and Management of COPD (GOLD) of 2025 highlighted that COPD is the third leading cause of death in the world, which for the majority of patients is not only preventable, but often derives from recreational habits such as smoking (as well as from exposure to pollution and the work or living environment) [2]. The mortality rate of this chronicity is around 6–7% in the world [3,4].

Another chronic condition is chronic heart failure (CHF), which accounts for approximately 1.1–5.5% of the global population, with a one-year survival rate of 4–45% [5,6,7]. CHF is a multifactorial syndrome, the causes of which can range from a previous myocardial infarction to non-rheumatic calcific aortic valve disease, non-rheumatic degenerative mitral valve disease, and myocarditis; all of these events alter the cardiac structure over time, resulting in impaired contractile function [8].

In these chronic diseases, we can find some recurrent events that link these syndromes: cognitive decline (CD) and diaphragmatic dysfunction (DD).

In patients with COPD, general memory and verbal ability, as well as learning and attention, are reduced by approximately 61%; this cognitive deficit correlates directly with the severity of the disease, which (hypoxemia and hypercapnia) can push the percentage of cognitive deficit up to 77% [9,10]. Mortality in the presence of COPD and CD increases the mortality rate almost fivefold compared to COPD without CD [11]. The mechanisms responsible for CD in patients with COPD remain elusive [10].

In patients with CHF, there is a direct relationship between CD, levels of N-terminal pro-B-type natriuretic peptide (NT-pro-BNP, a parameter that demonstrates cardiac contractile decline), and disease severity (left-ventricular ejection fraction, LVEF) [12]. The incidence of CD in patients with CHF is high (40–80%), with a 50% higher death rate compared to patients with CHF without CD [12,13]. The pathophysiological causes remain largely unknown [12].

In patients with COPD, a decline in diaphragmatic function (weakness and hypomobility) is present at every stage of the disease (100%), and correlates directly with disease severity and prognosis [14]. In patients with CHF, diaphragmatic dysfunction (weakness and hypomobility) is found at every stage of the disease (100%), and correlates directly with disease severity and prognosis [15].

The article briefly reviews the possible causes of CD in patients with COPD and CHF, diaphragmatic adaptations, and raises new considerations that may underlie the finding of cognitive decline in these two chronic syndromes. The hypothesis of implementing diaphragmatic respiratory rehabilitation (IMT) as a non-pharmacological tool to combat the onset of CD is proposed, possibly by improving the function of the cerebral fluid system, such as the glymphatic system and cerebrospinal fluid.

### 1.1. Chronic Obstructive Pulmonary Disease and Cognitive Decline 

**Figure 1:**
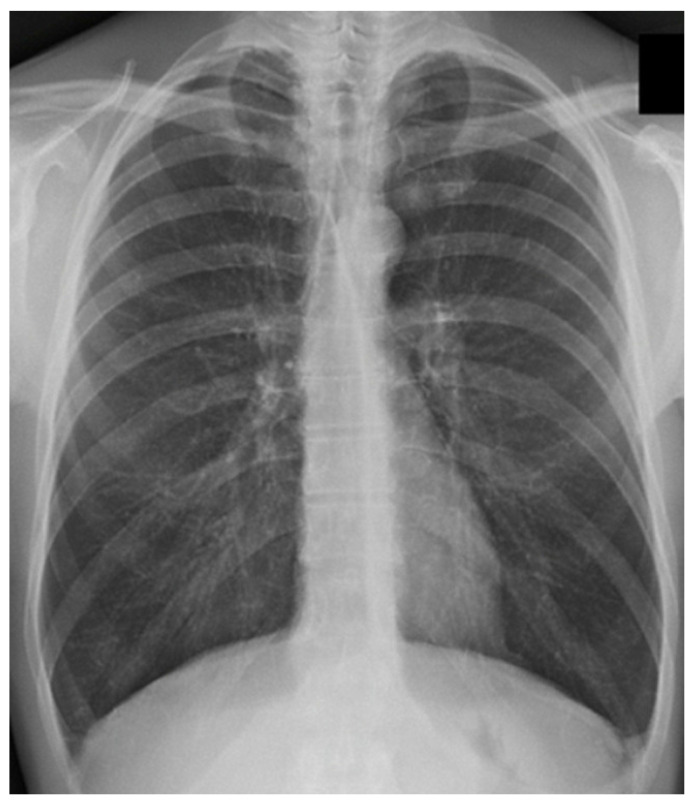
Chest x-ray of a COPD patient. The diaphragm is in a chronic inspiratory position (lung hyperinflation or hyperdistension) with a more flattened morphology. The constant, non-physiological position of the hemi-cupules causes a reduced elasticity of the lung parenchyma, an adaptation that parallels the chronicity of the disease. The photo was taken by Bruno Bordoni.

COPD is diagnosed by parameters derived from spirometry (post-bronchodilator forced expiratory volume in one second—FEV1), demonstrating airflow restriction, caused by chronic structural and functional alterations of the respiratory system (bronchitis, bronchiolitis, emphysema), although it is a preventable disease. Chronic symptoms such as productive or non-productive cough and dyspnea must be present, even if not all symptoms may be present in the same patient (heterogeneity of the disease) [2]. GOLD has four levels of severity: stage 1 or mild (FEV1 ≥ 80% predicted); stage 2 or moderate (FEV1 ≥ 50% predicted, < 80% predicted); stage 3 or severe (FEV1 ≥ 30%, < 50% predicted); stage 4 or very severe (FEV1 < 30% predicted) [2]. Furthermore, a fifth stage is also foreseen, defined as pre-COPD (or GOLD 0), where patients who do not have parameters of airway obstruction (PRISm or preserved ratio impaired bronchodilation), present clinical conditions (increased airway wall thickness) and/or habits (smoking) that can lead to the development of the disease (Figure 1) [16].

There are multiple risk factors and causes, deriving from differences in the phenotype (concomitant diseases) and the endotype (subtype of symptoms and conditions). From this consideration, the concept of gene (G)–environment (E) interactions that occur through the lifetime (T) (GETomics) was born [2,17]. GETomics is an acronym to highlight that the disease is the response deriving from multiple dynamic and interacting subjective factors and from daily life [17].

Smoking and exposure to pollutants or irritants are the fertile basis for the development of COPD; there are also genetic predispositions such as SERPINA1 gene deficiency, which decreases the α-1-antitrypsin response, altering the neutrophil elastase response [18].

Another classification of COPD, Airflow Obstruction by Ratio (STAR), has been proposed, which examines the relationship between FEV1 and forced vital capacity (FVC) (>0.60–<0.70, >0.50–<0.60, >0.40–<0.50, <0.40) [19]. STAR may be more sensitive to disease severity and mortality [20].

Patients with COPD are subject to frequent exacerbations (AECOPD), which increase the mortality rate, with a rate of 26.2% one year after the acute event, and a rate of 64.3% five years after the acute event [21]. AECOPD is divided into mild (patients treated with short-acting inhaled bronchodilators), moderate (patients treated with oral corticosteroids, short-acting inhaled bronchodilators, and antibiotics if necessary), and severe (patients hospitalized) [2].

Cognitive impairment can involve different brain areas, causing a decline in the processing functions of different afferent inputs (internal and external), and/or with altered efferent responses; in this patient population, CD can be mild or severe, up to dementia, with a four-fold higher incidence compared to non-COPD [22]. CD in COPD negatively affects memory, attention, and learning ability [22]. Some factors can be predictive in the detection of CD, such as age (>60 years), education (low), sedentary lifestyle, low engagement in intellectual activities, FEV1/FVC, low serum albumin values, presence of comorbidities, and social isolation [22,23,24].

Other factors that could explain CD are the lung–brain relationship and inflammation. Chronic exposure to irritants stimulates the production of mucin at the pulmonary epithelial level, a decrease in the efficiency of the cleaning epithelium itself, and a decrease in immunoglobulin A. These altered mechanisms will stimulate the bacteria present to enter the pulmonary epithelium, causing the production of macrophages, neutrophils, and lymphocytes (pro-inflammatory cells); in turn, these cells will stimulate the increase in leukocytes, creating a local and then chronic systemic inflammatory and oxidative environment [25]. Inflammatory substances thus produced, such as interleukin-6 and 1β, tumor necrosis factor-α, can cross the blood–brain barrier (BBB), damaging neural cells, stimulating further neuroinflammation [26]. Neuroinflammation would stimulate the activation of microglial cells, immune cells of the central nervous system; if these cells are activated to a non-physiological extent, oxidation increases and further gray and white matter is lost, leading to cognitive impairment [27].

Furthermore, hypoxia and hypercapnia resulting from chronic airway obstruction would determine a decrease in the production of oxygen-dependent enzymes, tyrosine hydroxylase and tryptophan hydroxylase, which are essential for the synthesis of some neurotransmitters such as dopamine and serotonin, respectively [28]. Alterations in dopamine and serotonin lead to the onset of CD [29].

CD in patients with COPD include multiple disorders and pathologies, such as mood disorders, dementia, Alzheimer’s disease, Parkinson’s disease, and anxiety disorder [22,30]. Currently, there is no effective approach to combat the presence of CD in this patient population, not to mention that a neurological pathology or mental disorder contributes to reducing the participation of patients in a pulmonary rehabilitation (PR) program [22]. With instrumental examinations (magnetic resonance imaging), a structural and functional alteration of the brain areas in COPD has been demonstrated, such as a decline in gray and white matter (atrophy), enlarged perivascular spaces (extracellular fluid stasis index), and decreased cortical thickness; in particular, such pathophysiological adaptations concern the prefrontal areas, Broca’s areas, parietal areas, motor areas, limbic areas, cingulate gyrus, midbrain, corona radiata, fornix, optic radiations, inferior longitudinal fasciculi, and the corticospinal tract, insula, and corpus callosum [31,32].

### 1.2. Chronic Heart Failure and Cognitive Decline 

**Figure 2:**
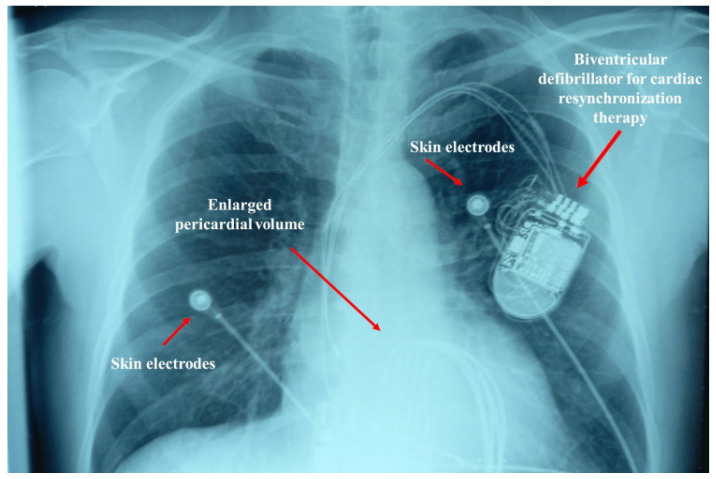
The image shows a patient with chronic heart failure. Cardiac dilation (and a low ejection fraction) is noted. The increase in thoracic space caused by the heart places a strain on the contractile capacity of the diaphragm.

CHF results from non-physiological cardiac alterations that cause an imbalance between the peripheral oxygenation demand and the heart’s working capacity, with pulmonary and/or systemic congestion [33]. The American Heart Association (AHA), the American College of Cardiology (ACC), and the Heart Failure Society of America (HFSA) classify patients with CHF into four distinct categories, based on contractile function (LVEF): preserved LVEF (HFpEF ≥ 50%); mildly reduced (HFmrEF 41–49%); reduced LVEF (HFrEF ≤ 40%); improved LVEF (HFimEF starting from a value of ≤40% and improving by approximately ≥ 10%) [34,35] (Figure 2).

In Europe, the European Society of Cardiology (ESC) divides CHF patients into three types, always based on the percentage of ejection frequency: HFpEF (LVEF ≥ 50%); HFrEF (LVEF ≤ 40%); HFmrEF (LVEF 41–49%) [36]. The severity of the disease is also assessed by the percentage of detection of N-terminal pro-brain natriuretic peptide (NT-pro-BNP, ≥ 125 pg/mL) or brain natriuretic peptide (BNP, ≥ 35 pg/mL), according to the indications of the American guidelines [37]. ESC suggests making differentiations based on the presence or absence of atrial fibrillation. In the latter case, the clinician should be alerted if blood NT-pro-BNP values are higher than 365 pg/mL, and BNP values higher than 105 pg/mL [37]. Another value that has recently acquired importance for risk stratification is the echocardiographic analysis of the behavior of the left atrium, in particular the peak atrial longitudinal strain (PALS). PALS would allow for better identification of diastolic dysfunction, and as a prognostic value independently of LVEF [38].

In this type of patient, episodes of worsening occur, requiring an increase in the therapeutic approach, possible hospitalization, and worsening of symptoms (peripheral and central congestion, dyspnea and orthopnea, decreased ability to perform physical activity) [39].

There are multiple causes that lead to CHF, such as ischemic cardiac events (risk of development of 13–62%), and the presence of various previous morbidities, such as obesity (risk of development of 12.8%, 29.9%), cancer (risk of development of 3.96–6.68%), diabetes mellitus (risk of development of 3–12%), hypertension (risk of development of 10–59%), chronic kidney disease (risk of development of 18.3%), COPD (risk of development of 15.6%), arrhythmia (risk of development of 2–48.7%); the risk percentages increase if these pathologies are added together, and if smoking, a sedentary lifestyle and advanced age are present [40,41,42,43,44].

Reduced cardiac function alone in CHF, or structural alterations of the vessels or the autonomic system (hypertension), do not explain the brain atrophy found in patients (decreased perfusion) [45]. An MRI of HFrEF patients has demonstrated cortical atrophy (frontal, temporal, and parietal lobes, middle-superior and post-central and anterior gyrus, prefrontal cortex, occipital cortex, insular and hippocampal area, thalamus and fornix, putamen and cerebellum, caudate nuclei, forebrain and brainstem, mammillary bodies, raphe), and a changed cortical morphology (increased curvature as in patients with Alzheimer’s disease) due to a decline in white matter (gray matter decline) [45,46,47]. This remodeling lead to CD and dementia and are not necessarily related to advanced age [48]. Similar brain changes are found in patients with HFpEF [48]. The condition that leads to CD without a direct link to brain oxygenation is known as cardiocerebral syndrome [48].

One reason for cortical and subcortical atrophy in CHF is elevated cortisol levels. High levels of cortisol (due to dysfunction of the renin–angiotensin–aldosterone system) can prevent neurogenesis by decreasing the amount of gray and white matter [48]. Elevated epinephrine levels may damage the central nervous system indirectly by elevating glucose levels and increasing oxidation processes, negatively impacting brain functions, such as memory [48]. A constant state of systemic inflammation as in CHF is another factor that may cause damage to the nervous system. Patients with CHF and CD have increased levels of pro-inflammatory cytokines (interleukin-6—IL-6, tumor necrosis factor—alpha—TNF-α), and high levels of total plasma homocysteine that stimulate apoptosis of neural cells, with brain atrophy [48].

Another cause of brain atrophy in these patients could be related to low levels of vitamin B1 or thiamine (acts as a coenzyme for neural cellular energy), particularly in hospitalized CHF patients (but not in non-hospitalized CHF patients) [49,50]. Other causes are related to thrombus formation due to altered blood rheology in these patients, which causes small ischemic damage [51]. Altered autonomic reflexes (peripheral chemo-baroreceptors and inadequate central sympatho-vagal balance) can cause cerebral vascular spasms, leading to hypoperfusion and brain damage [52]. Hypoperfusion is a common event in CHF, as demonstrated by transcranial Doppler examination, with increasing hypoperfusion over the course of 12 months. There could possibly be a smaller number of collateral vessels and lower perfusion due to atheromatous stenosis or arterial stiffness (vascular cognitive impairment) [53]. Hypoperfusion does not correlate, however, with the percentage of LVEF [53].

Another cause that could lead to CD is proteotoxicity. The three-dimensional shape of a protein determines its function; an alteration of the mechanisms underlying the biological process of protein folding (protein misfolding) is one of the causes that contribute to the onset of neurodegenerative diseases [54]. An increase in misfolded proteins transported from the endoplasmic reticulum into the cytoplasm (where they are poly-ubiquitylated and degraded) will lead to accumulation, with the formation of inclusion bodies; the latter will cause proteotoxicity and neural cell death [55]. This mechanism of accumulation of toxicity could occur, albeit rarely, in patients with CHF [56].

As with COPD, advancing age and microvascular changes can accelerate the decline in brain function and structure, such as white and gray matter atrophy [57]. The myelin sheaths begin to show loss of structure and efficiency from the age of forty, and gray matter begins to decline in volume after the age of twenty; the amount of serotonin and dopamine from adulthood begins to decline, with decreased capacity for synaptogenesis, paving the way for CD [57].

In chronic diseases such as CHF and COPD, the co-presence of pathologies such as diabetes (metabolism alteration), atrial fibrillation (formation of microemboli), chronic kidney disease (accumulation of uremic toxins), and multiple instances of ingesting different drugs (summation of side effects) increases the percentage of CD detection [57,58].

A mechanism that allows the cerebral pathways to be kept clean, eliminating waste products, correctly managing interstitial pressures, and the various molecular exchanges is the glymphatic system (a mixture of interstitial fluids and cerebrospinal fluid) [59]. A non-physiological alteration of this system causes neurodegenerative diseases [60,61]. Some factors determine the movement of the glymphatic flow (brain-wide fluid transport pathway), such as arterial pulsatility (deriving from the intervention of the autonomic system), heart rate, and respiration [62]. In pathologies such as CHF and COPD, the autonomic system is dysfunctional (greater intervention of the sympathetic system), and the cardiovascular system is compromised, as observed in the findings of DD [63,64,65,66]. We can strongly assume a functional decline of the glymphatic system in the presence of COPD and CHF.

### 1.3. Glymphatic System

The glymphatic system is a maze of leptomeningeal tunnels or perivascular spaces (Virchow–Robin) delimited by astrocyte endfeet. Numerous aquaporin-4 (AQP4) water channels are present on astrocytes, which promote the passage of fluids (cerebrospinal fluid and interstitial/extracellular fluids) towards the brain parenchyma, thanks to the presence of gap junctions between the astrocyte cells and their endfeet [67]. Fluids can move through diffusion (for small molecules) and convection (for larger molecules) [68].

**Figure 3:**
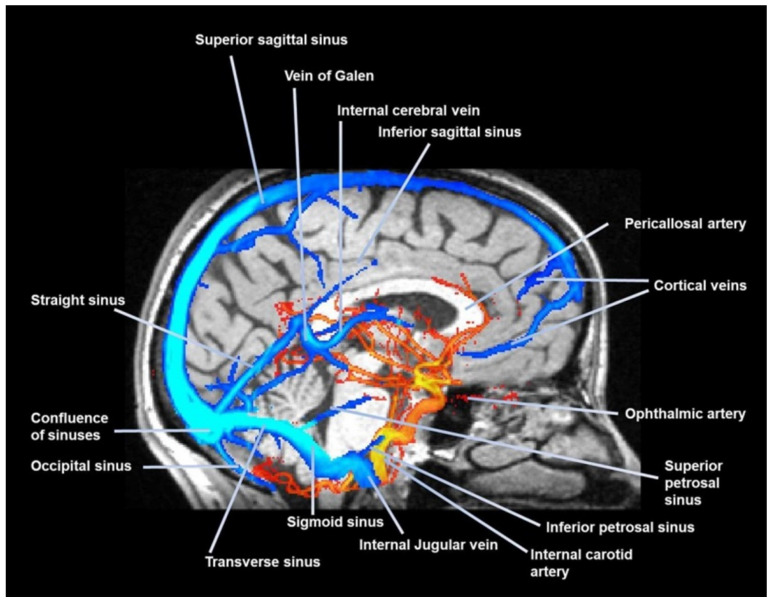
A sagittal view with magnetic resonance imaging. The image shows the venous sinuses and some arteries of the skull in a healthy subject. The photo was taken by Bruno Bordoni.

Glymphatic fluid and cerebrospinal fluid (CSF) can return to the Virchow–Robin space following the arterial pathways, in a motion opposite to the direction of arterial blood, via the vasomotion of cerebrovascular smooth muscle cells [69]. This arterial filtration mechanism allows the central nervous system to be cleared of waste solutes and metabolites, such as beta amyloid (Aβ) and tau protein [68]. In summary, these spaces serve as the lymphatic system of the brain. A portion of the CSF also follows other pathways, such as the dural lymphatics from the subarachnoid space; from here, CSF can travel to the bone marrow of the cranial bones, and/or to the cranial/extracranial nerve pathways, reaching the ethmoid plate, to enter the nasal mucosa and, like the fluids within the glymphatic system, will flow towards the cervical lymph nodes [68,70]. CSF can be reabsorbed by the glymphatic system several times along its path [59] (Figure 3).

Fluid flow within the glymphatic system is facilitated by vasomotion (oscillation of approximately 0.1 Hz, and a propagation velocity of approximately 0.2–2 mm/s), which is induced by the autonomic nervous system and, in particular, by the parasympathetic nervous system [71]. The latter promotes vasodilation and proper self-regulation of vasomotion [72,73]. Vasomotion is also influenced by surrounding neurons (intrinsic movements and oscillations or electromotive force), with hemispheric differences depending on the callosal projections, and the amount of oxygen, potassium, and calcium [72].

In animal models, AQP4 activity influences the diurnal/nocturnal movement of the glymph (CSF and interstitial/extracellular fluids) and of the CSF [67]. CSF, like glymph, has peak quantities during the night hours and will probably use preferential pathways depending on the presence of light and darkness [67]. There is a close relationship between CSF and glymph, as well as the motion that allows its distribution.

In healthy subjects, the heartbeat provides approximately 15–25% of the force capable of moving CSF/glymph within the central and peripheral nervous system (spinal cord), with a variable force depending on the brain area (perivascular pumping), with centrifugal stimuli during systole; inspiration stimulates a centripetal movement, with greater amplitude and force [74,75,76]. Breathing helps the glymphatic system to remove metabolic waste, to a greater extent than the heartbeat [74,76,77]. If the heartbeat is rapid, the movement of the glymph appears to be smaller [77].

Glymph/CSF can also be absorbed and then diluted by following the leptomeningeal venous paravascular spaces (delimited by astrocyte endfeet), as the capillaries and venules neighboring then exiting the skull with the venous pathways [78]. Probably, breathing acts with greater emphasis on the venous paravascular spaces (they expand with inspiration because the veins collapse), since the heart acts mainly on the Virchow–Robin spaces [79,80]. Figure 3. Glymphatic and CSF movement is not linear, but chaotic (apparently).

During the cardiac cycle in healthy subjects, the brain mass undergoes a caudal–cephalic movement of approximately 0.43 mm; with systole, the brain mass and spinal cord undergo a cephalic movement, while with diastole an opposite movement is observed [81,82]. The extent of the displacement varies depending on the brain region, where the cerebellum area has an average movement of 0.067 mm, and the brainstem area has an average movement of 0.117 mm; the spinal cord has an average cranio-caudal displacement of 0.230–0.330 mm, always in healthy subjects [82]. This mechanism allows cerebral fluids to flow adequately [83,84]. With forced inspiration in healthy subjects, the cranial and caudal movement (with expiration) has an average of 2–3 mm; with the increase in venous pressure during inspiration, the brain mass and cerebral fluids compensate with a cranial direction [83]. Increased cranial blood pressure is compensated by caudal movement [83]. Breathing moves cerebral fluid and brain mass/spinal cord more slowly but allows for a greater amount of fluid movement; conversely, heartbeat accelerates craniocaudal movement of brain mass/spinal cord, while displacing less fluid [84]. Deep inspiration “inhibits” the force of the heartbeat (decreases cardiac stroke volume) in moving cerebral fluid, i.e., diaphragmatic force becomes the most representative in this movement/displacement mechanism [84]. Voluntary apnea limits CSF/glymph movement [74].

### 1.4. The Cerebrospinal Fluid and the Glympatic System 

The glymphatic system guides CSF within the central nervous system between the different periarterial and perivenous spaces [85]. Glymph (extracellular fluids) and CSF move with the same mechanisms and pathways.

If the glymphatic system is dysfunctional and/or there is a decrease in AQP4, with reduced glymph/CSF drainage, metabolites and highly phosphorylated proteins (Aβ, tau protein) accumulate, leading to neurodegenerative diseases, from dementia to Parkinson’s disease [66,78,85].

We can state that a cardio-respiratory dysfunction will negatively impact the glymphatic system. Some initial confirmations come from an animal model of CHF. In this latter pathological condition, cerebral fluids increase, but not the clearance of metabolites; that is, a dysregulation of brain fluid dynamics occurs, which leads to CD in its various pathological forms [86]. The glymphatic system should become a target of greater therapeutic attention in patients with CHF [75,87]. A recent study evaluated the dynamics of the glymphatic system in patients with atrial fibrillation compared to healthy subjects. Although the patient group was limited (13), the authors highlighted a dysregulation of the glymphatic/CSF cerebral fluid mechanisms; this disruption of the fluid movement patterns is the basis for the detection of future cerebral degenerative pathologies [88]. It increases the stasis and pressure of these fluids towards the central nervous system, creating an inflammatory environment [89].

Regarding COPD, we have only indirect data. Common alterations in cerebral vascular structure and function (altered cerebral blood flow response to arterial carbon dioxide (CO2) levels) are found, as in various CD, and with larger perivascular spaces [90,91,92]. The increase in the size of these spaces is one of the causes that accelerates the onset of Alzheimer’s disease [93]. Hypertension in COPD patients is common (54.2%), and we know that this clinical condition alters the behavior of CSF (increases in volume but with reduced clearance), leading to CD [94,95,96]. We can strongly hypothesize that the cerebral fluid system is dysfunctional in patients with COPD.

This stasis changes the composition of the fluid system’s macromolecules, which reflect the local inflammatory status, decreasing the system’s ability to eliminate accumulated metabolites [97].

In patients with COPD and CHF, systemic inflammation, including neuroinflammation, is present [98,99]. Systemic inflammation activates microglial cells, which will change morphology (morphologically “amoeboid”), the most important cells in the immune response of the central nervous system, which will induce cyclooxygenase-2-dependent neuroinflammation [92,100]. The latter will increase the levels of superoxide anion, leading to a chronic oxidative environment, altering the brain and vascular structure [92,100]. Active glial cells can also promote pro-inflammatory cytokines, creating a self-perpetuating cytotoxic inflammatory cycle, and/or produce pro-apoptotic substances [100].

There are multiple factors that alter this delicate mechanism of fluid movement in an already inflamed environment, and this altered pattern will highly likely lead to CD.

Another factor that links these two chronic diseases and the alteration of cerebral fluid function and movement is advanced age. Advanced age is common in COPD (10.7% of patients are elderly in the US), and in patients with CHF (the risk percentage of finding is over 10% above 70 years) [101,102]. Aging slows the proper flow of the glymphatic/CSF system, and AQP4 decreases, increasing systemic inflammation [103]. The diaphragm is weaker in elderly subjects (transdiaphragmatic pressure declines by approximately 20–41%) [104]. Aging declines the diastolic functional capacity of the left ventricle, and there is an increase in left ventricular thickness, making tachycardia and atrial enlargement more likely [105]. It is probable that the glymphatic/CSF system is more functionally impaired in elderly COPD and CHF patients, considering that the diaphragm and cardiac muscle have less strength.

Another element found in patients with COPD and CHF is stress. Stress increases smoking and increases the possibility of recording an acute phase in COPD fourfold, with a detection rate of 14–25% [106,107]. The percentage of stress perceived by patients with CHF is approximately 60%, and stress can accelerate the progression of the disease and CD [108,109]. In animal models, stress reduces the effectiveness of cerebral fluid movement, decreases the number of AQP4, and decreases the number of proteins that allow for its positional stabilization (agrin, laminin, dystroglycans) [68,110]. The reasons for this are linked to increased sympathetic activity and excess production of glucocorticoids, leading to CD [110].

In an inflamed environment, glymph and CSF transport inflammatory and immune substances (macrophages, neutrophils, monocytes, cytokines). It has recently been demonstrated that this fluid system comes into contact with the bone marrow of the cranial bones, through sub-millimeter channels (40–150 µm wide in humans) and paravascular spaces, transporting what the immune system produces. The cranial bone marrow acts as an immune sensor. The hematopoietic activity of the bone marrow in contact with glymph and CSF full of inflammatory and immune substances will produce lymphocytes, myeloid cells, and leukocytes [111,112,113]. The latter will return through paravascular spaces to the brain parenchyma, creating a closed circle of neuroinflammation, causing CD and neurodegenerative diseases over time [113].

The forces that allow for proper flow and drainage of the glymph/CSF derive from the heartbeat, primarily from respiratory activity, and, to a lesser extent, from the autonomic system. Impaired function of these three factors will negatively impact cerebral fluid flow, with detrimental consequences for the proper functioning of the central nervous system. Of these three factors, the diaphragm influences both cardiac function and autonomic response. In the presence of COPD and CHF, the diaphragm undergoes non-physiological adaptations.

### 1.5. Assessments for Dementia Detection

Dementia is the seventh leading cause of mortality and morbidity in the world [114]. The clinician has at his disposal some investigative tools for the classification of dementia: the Folstein Mini-Mental State Examination (MMSE), the Montreal Cognitive Assessment (MoCA), the Diffusion Tensor Imaging Along the Perivascular Space (DTI-ALPS). MMSE (first published in 1975) is a short test, very widespread in the field of dementia screening, through which the clinician can evaluate, with a certain degree of reliability, the neuro-cognitive and functional state of a patient. The test is composed of eleven very simple questions and small graphic tasks, which allow the clinician to probe different aspects of brain function (orientation, memory, attention, calculation capacity, the ability to recall certain acquisitions, language) [115].

MoCA (devised in 1996 by Dr. Ziad Nasreddine) involves the clinician asking the patient to perform neuromotor and comprehension tasks (12 subtasks) and has the advantage of intercepting early signs of dementia. The test evaluates functions such as visuospatial abilities, orientation, object naming, attention, expressed language, abstraction capacity, and memory. The score varies from zero for a deficient performance to 30 for a perfect performance [116].

DTI-ALPS is a non-invasive medical diagnostic tool introduced in 2017 for the evaluation of the glymphatic system; it uses diffusion tensor images to average the diffusion of water into the interstitial spaces and the diffusion of water into the perivascular space. An alteration in the distribution of water assessed with this tool (lower ALPS-index) indicates the presence of dementia, such as Alzheimer’s disease, Parkinson’s disease, and other disorders [117]. Another instrumental method for evaluating the glymphatic system is the use of MR cisternography and the intrathecal or intravenous administration of gadolinium-based contrast agents (GBCAs), particularly for the more superficial area of the brain [117]. Another methodology used in the clinical setting for the evaluation of cerebral neural fluid movement is arterial spin labeling (ASL), with good sensitivity in assessing perivascular clearance [117]. Dynamic positron emission tomography (PET) is another strategy for evaluating the glymphatic system and identifying possible cognitive impairments [117].

### 1.6. COPD and the Diaphragm

The diaphragm affects cardiac and autonomic function. Muscle fibers have lower protein content and are shortened (inspiratory posture) [63,118]. The diaphragm is in a lower position than in healthy subjects, and this condition reduces the radius of the diaphragm in the apposition zone, placing it at a biomechanical disadvantage [119]. Transdiaphragmatic pressure (Pdi) decreases by approximately 40%; the force expressed by the diaphragm is approximately 35% lower than in healthy subjects [120]. Magnetic resonance imaging (MRI) has shown that in COPD, the diaphragm moves the two hemi-cupules asynchronously, and this phenomenon is more significant in more severely affected patients (GOLD-IV) [121]. The phrenic nerve shows neuropathic damage, particularly the left nerve; the greater the lesion, the greater the presence of hyperinflation [63]. The movement capacity of the diaphragm is globally reduced, and non-physiological adaptations are found in all patients with COPD, regardless of the stage of the disease [118,122].

The fibers undergo a phenotypic shift, with an increase in red/aerobic fibers and a decline in white/anaerobic fibers; the fibers show signs of atrophy and fibrosis [63]. A decline in heavy-chain myosin of approximately 30–50% is recorded, with cytotoxic accumulations of intracellular calcium due to dysfunction of the sarcoplasmic reticulum [63,123]. Other sarcomeric proteins undergo a decline in function and quantity, such as nebulin and titin, making the fiber more sensitive to microtrauma [63,124]. Sarcomeric proteins lose their correct serial arrangement, collagen increases; the sarcomere is shorter by approximately 28% [63]. Chronic inflammatory processes, oxidation and apoptosis are present within the diaphragm, the levels of which reflect the severity of the disease stage [63,118]. The diaphragmatic fiber has a lower repair capacity, with increases in myostatin and a decrease in the repair capacity of satellite cells [63].

Not only does DD in COPD (and CHF) alter the patient’s symptomatic picture, but it alters the autonomic response towards a greater sympathetic response [125,126,127].

Inhalation creates pressure gradients for proper venous return to the right atrium, without stressing the right-chamber portion of the heart, thanks to the force of the preload (myocardial stretching) and the right systolic output [65,128]. Always during diaphragmatic contraction, inspiration manages the ventricular afterload of the left portion, as well as the aortic diastolic pressure gradient; with the act of expiration, the left cardiac chambers are filled, allowing for a reduction in the aortic diastolic pressure gradient, with the left ventricular systolic output volume increasing. The diaphragm heavily influences cardiac function, influencing its systolic and diastolic pressures [65,128]. If the presence of DD persists, the sympathetic system takes over, altering systemic and central hemodynamic modulation, and cardiac chronotropy [125,127].

### 1.7. CHF and Diaphragm

The pathophysiological adaptations that the diaphragm undergoes occur rapidly upon the onset of the disease [65,129]. The neuromotor framework undergoes a functional decline, with signs of partial denervation; as a result, the force expressed by the diaphragm is reduced by approximately 15–30%, as is the contraction speed, which is reduced by approximately 20–30% compared to healthy subjects [65,129]. The fiber undergoes atrophy, with a decrease in the protein titin and myosin/actin, in both aerobic and anaerobic fibers; the percentage of connective tissue and fibrosis increases [65,129,130]. Contractile capacity is reduced, movement is reduced, and it is more susceptible to microtrauma, with a reduction in overall thickness and a decrease in fatigue resistance [131,132]. There is a direct relationship between CHF, DD, and FEV1 values, blood–brain natriuretic peptide levels, and disease severity [133]. The diaphragm plays a key role in the symptoms and clinical evolution of the disease [65,133].

DD increases the incidence of anxiety and depression (CHF and COPD), which are potential gateways to CD and dementia [125,126,127,134,135].

### 1.8. Inspiratory Muscle Training (IMT)

Considering the data reported in the literature, a factor that could become decisive in improving the glymphatic system (glymph and CSF) and decreasing the occurrence of CD in patients with COPD and CHF is the diaphragm. The latter can be trained through inspiratory muscle training (IMT), which is considered in European and American guidelines for patients with these chronic diseases [2,134,135,136,137,138].

IMT is a rehabilitation approach based on overcoming certain resistances during inspiration, using devices (different depending on the manufacturer) with different forces depending on the metabolism that the patient needs to train. The forces to be overcome are calculated on the maximal inspiratory pressure (PImax), which will be lower than 50%, with high repetitions and sets if the metabolism that one wants to involve is aerobic for the red fibers, or a PImax around or higher than 50%, with fewer repetitions and sets if the metabolism that one wants to train is anaerobic for the white fibers [2,136,137,138]. Improving or delaying pathophysiological adaptations of the diaphragm through IMT means increasing or maintaining the range of motion during respiratory acts, creating pressures in the body that are fundamental for health.

Current data show that aerobic physical activity in healthy or neurodegenerative human and animal models improves glymphatic flow, thus improving CD [139,140]. In animal models, aerobic physical activity improves the clearing capacity of the glymphatic system, increases the number of AQP4, and there is a lower accumulation of amyloid plaques and less microglia activation, suggesting that physical activity can counteract brain functional decline [68].

IMT alone or in combination with a rehabilitation regimen in patients with CHF can improve multiple functions and parameters, such as distance covered in the six-minute walking test (6MWT), expressed diaphragmatic strength and thickness, improved oxygen consumption, and quality of life (HRQoL) [141]. IMT for patients with COPD demonstrates improvements in several respiratory parameters, such as FEV1 and forced vital capacity (FVC), as well as functional improvements such as 6MWT and HRQoL [142].

To the authors’ knowledge, there is no study in the literature that has evaluated IMT in animal or human models with respect to cerebral fluid function, and with respect to CHF and COPD and in the presence of CD. We can state that general physical activity involves the respiratory system (and consequently the glymphatic system), but we cannot state whether IMT alone or with general training (rehabilitation for these chronic patients) can create further improvement of the glymphatic system in patients with COPD/CHF and CD.

There are multiple indirect reasons for the usefulness of improving diaphragmatic function and excursion through IMT, given the clinical conditions found in this type of patient and glymphatic/CSF function and CD.

Reduced diaphragmatic excursion causes sympathicotonia in patients with CHF, creating a metabolic and humoral cascade that is extraordinarily important for the development and progression of the disease. Diaphragmatic targeting should not only be included in a rehabilitation regimen, but as a constant therapeutic tool [143]. Likewise, the presence of DD in patients with COPD leads to a predominant increase in the sympathetic system [63].

IMT in patients with CHF, measured by microneurography, can reduce the activity of the sympathetic nervous system; similarly, in COPD patients, IMT reduces the excitation of the sympathetic system [144,145]. A reduction in the adrenergic system can improve the space in which glymphatic fluids/CSF can flow, improving the efficiency of clearance and transport [77]. It is possible to hypothesize that by increasing the excursion of the diaphragm and improving the parasympathetic response, benefits could be obtained through this system and the possible onset of CD could be postponed in this patient population.

More effective breathing can improve blood pressure values in CHF [146]. An exercise that falls within diaphragmatic stimulation, but without a resistance to overcome, is yoga breathing. The latter involves the patient’s awareness of the act of breathing, making the breath deeper; an improvement in breathing patterns with a yoga approach improves diastolic blood pressure in COPD [147]. We have not found in the literature any studies with IMT and COPD that have observed changes in blood pressure. Certainly, lowering blood pressure improves the glymphatic system [96].

IMT can reduce some blood parameters related to systemic inflammation and cardiac dysfunction, such as N-terminal fragment of the brain natriuretic peptide, C-reactive protein, soluble Fas (soluble form of the Fas receptor CD95/Apo-1), and human soluble tumor necrosis factor receptor I [148,149]. More recent data with a larger number of patients are lacking to further support these latest results. More recent data concern the area of chronic lung disease. Respiratory muscle training has statistically valid results in improving strength (PImax), diaphragm movement and several ventilatory parameters (FEV1, FVC), and performance (6MWT), as well as improving dyspnea and HRQoL [150]. With IMT, glutathione (antioxidant) levels increase, as well as total antioxidant capacity, while lipid peroxidation (malondialdehyde) and pro-inflammatory cytokine (IL-6, TNF-α) levels decrease [150]. One explanation for the diaphragmatic ability to induce an anti-inflammatory environment is the stimulation of the parasympathetic system [130]. The parasympathetic system counteracts the action of the sympathetic system (the release of catecholamines that activate immune cells), via the anti-inflammatory cholinergic pathway (intervention of the vagus nerve, cranial nerve X) [151].

IMT improves the sense of dyspnea in patients with CHF and COPD [141,152]. This adaptation has a positive impact in reducing stress and anxiety levels, as well as depression [107,153,154,155].This improvement in psychological and emotional state should delay the decline in glymphatic/CSF system function and the finding of CD in this patient population. We know that depression and altered emotional states induce CD in patients with CHF and COPD [156,157]. We know that depression alters the function of the glymphatic/CSF system, creating a closed cycle of pathological alterations [158].

**Figure 4:**
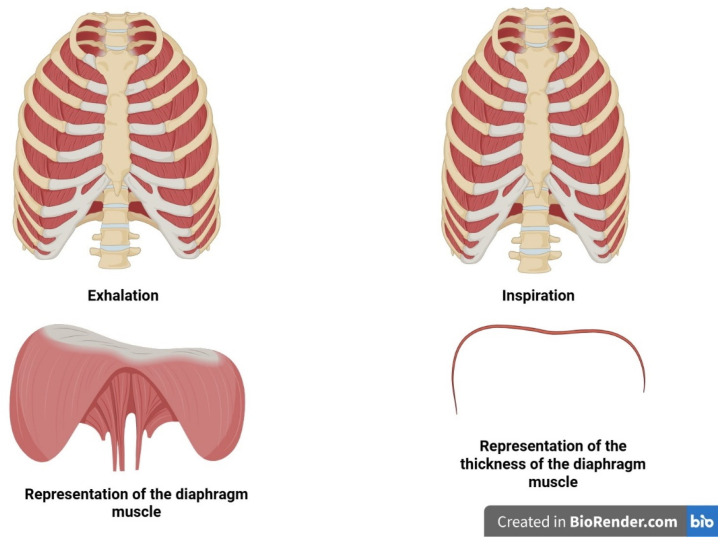
The image shows a schematic representation of how the rib cage moves during exhalation and inhalation (top right and left, respectively), and the bottom shows a schematic representation of the diaphragm muscle morphology (bottom right) and its thickness (bottom left). The image was created by Bordoni Bruno with a subscription to Biorender.com.

There is a relationship between the presence of inflammation, depression, stress, anxiety, predominant sympathetic nervous system, diaphragmatic dysfunction, and glymphatic/CSF dysfunction, with the occurrence of CD in patients with CHF and COPD. Data suggest that IMT can improve several parameters related to the clinical conditions listed above, potentially positively impacting cerebral fluid function, delaying the onset of CD and, consequently, improving morbidity and mortality in patients. (Figure 4)

### 1.9. Future Challenges

Research should focus on the relationship between the glymphatic/CSF system and CD in patients with CHF and COPD, both from a rehabilitation and pharmacological perspective. We do not know which IMT parameters are best suited for this patient population and for their subtypes. We do not know the optimal load to overcome during inspiration, how many repetitions, how many sets, and how many rest periods should be considered between sets and between sessions, nor how to align IMT with other rehabilitation sessions (cycling and muscle strengthening exercises). We do not know how to modulate training intensity, whether to follow a linear approach (constantly increasing resistance) or a nonlinear approach (planning sessions in which the training intensity is reduced), to avoid a potential decline in performance related to overtraining syndrome.

Furthermore, clinicians, such as cardiologists and pulmonologists, should pay greater attention to the diaphragm muscle, which plays an extraordinary role in bodily health but is too often underestimated clinically. Research should be conducted with IMT parameters that are as similar as possible to obtain more consistent and non-contradictory clinical responses.

Finally, GOLD 2025, as well as the American Heart Association (AHA), the American College of Cardiology (ACC), and the Heart Failure Society of America (HFSA), should include greater links to diaphragm muscle dysfunction and the presence of dementia.

## 2. Conclusions

COPD and CHF are chronic diseases, with COPD being the third leading cause of death worldwide, while CHF accounts for approximately 1.1–5.5% of the global population, with a one-year survival rate of 4–45%. These chronic diseases have multiple causes, and they share some common clinical symptoms, such as diaphragm dysfunction (DD) and cognitive decline (CD), which, in turn, increase patient mortality and morbidity. This article briefly reviewed the possible causes of CD in patients with COPD and CHF, including diaphragmatic adaptations, raising new considerations that may underlie the finding of CD in these two chronic syndromes. The possibility of implementing IMT as a non-pharmacological tool to combat the onset of CD was raised, thanks to the potential improvement in the function of the cerebral fluid system, such as the glymphatic system and cerebrospinal fluid. Further research should be conducted to ascertain the usefulness of IMT compared to the glymphatic system for improving cognitive decline in these patients.
